# Atogepant for migraine prevention: a meta-analysis of safety and efficacy in adults

**DOI:** 10.3389/fneur.2024.1468961

**Published:** 2024-09-27

**Authors:** Adarsh Raja, Rabia Asim, Muhammad Hamza Shuja, Sandesh Raja, Tazheen Saleh Muhammad, Simran Bajaj, Abdul Hadi Ansari, Hamza Ali, Iffat Ambreen Magsi, Muhammad Hammad Faridi, Hamza Ali Hasnain Sheikh, Muhammad Junaid Imran, Muhammad Ahmed, Muhammad Sohaib Asghar

**Affiliations:** ^1^Department of Internal Medicine, Shaheed Mohtarma Benazir Bhutto Medical College Lyari, Karachi, Pakistan; ^2^Department of Internal Medicine, Dow University of Health Sciences, Karachi, Pakistan; ^3^Department of Internal Medicine, Shaheed Mohtarma Benazir Bhutto University, Larkana, Pakistan; ^4^Department of Internal Medicine, AdventHealth, Sebring, FL, United States; ^5^Department of Internal Medicine, Mayo Clinic, Rochester, MN, United States

**Keywords:** atogepant, CGRP, migraine, headache, meta-analysis

## Abstract

**Background:**

Migraine is a neurological condition marked by frequent headaches, which tends to be accompanied by nausea and vomiting in severe instances. Injectable therapies for migraine, such as monoclonal antibodies that target calcitonin gene-related peptide (CGRP), have proven to be effective and safe. While various oral drugs are available, none have been developed for migraines. Patients prefer oral therapies because they are easier to use, making atogepant, an orally accessible small-molecule CGRP receptor antagonist, a possible alternative.

**Objectives:**

This systematic review and meta-analysis compared the safety and effectiveness of atogepant with placebo in treating migraine.

**Methods:**

Adhering to the PRISMA guidelines, we meticulously gathered randomized controlled trials (RCTs) from databases including the Cochrane Library, PubMed, Science Direct, and ClinicalTrials.gov. Studies comparing atogepant with placebo and reporting monthly migraine days (MMDs) as the primary outcome along with secondary outcomes such as monthly headache days and acute medication use days were included. Two independent reviewers conducted the data extraction and quality assessment. Statistical analyses were carried out using RevMan, utilizing risk ratios for dichotomous outcomes and mean differences for continuous outcomes, and a random-effects model.

**Results:**

Our primary outcome was the change in MMDs over 12 weeks, which showed a significant reduction with atogepant at dosages of 10, 30, and 60 mg. Secondary outcomes, such as monthly headache days, proportion of patients achieving a ≥ 50% reduction in MMDs, acute medication use days, and patient-reported outcomes, consistently showed that atogepant outperformed placebo, highlighting its effectiveness in reducing the migraine burden.

**Conclusion:**

Higher doses of atogepant are more effective in lowering migraine and headache-related days and increasing quality of life metrics. However, this is accompanied by an increased incidence of adverse events, suggesting the need for careful dose optimization to balance the benefits and risks.

**Systematic review registration:**

https://www.crd.york.ac.uk/PROSPERO/display_record.php?RecordID=563395. Unique Identifier: CRD42024563395.

## Introduction

Migraine is a persistent brain disorder characterized by recurring attacks that involved throbbing headaches on one side of the head along with symptoms such as vomiting, nausea, phonophobia, and photophobia. Individuals often experience episodic migraine, which can lead to chronic migraine. The International Classification of Headache Disorders, 3rd Edition (ICHD-3) describes episodic migraine as less than 15 headache days monthly and is experienced by 91–93% of individuals with migraines (1). Chronic migraine, which affects 1–2% of the world’s population, is defined as a minimum of 15 headache days per month, with at least 8 of them fitting the migraine criteria (2).

Calcitonin gene-related peptide (CGRP) is involved in the development of migraines, with studies indicating elevated systemic levels during migraine episodes (3, 4). Inhibitors targeting the CGRP pathway have emerged as a new strategy for preventing migraines and are recommended for approximately 40 percent of patients who experience frequent or severe episodes, particularly those who do not respond adequately to other preventive treatments. Despite the widespread prevalence and significant clinical impact of migraine, there is a dearth of rigorous clinical trials and effective pharmacological treatments. Current oral preventive therapies include *β*-blockers, tricyclic antidepressants, angiotensin receptor antagonists, and antiepileptics, none of which was originally developed specifically for migraines (5). Poor efficacy and tolerability often lead to failure and discontinuation of migraine treatments (6, 7).

Although several injectable monoclonal antibodies (erenumab, fremanezumab, galcanezumab, and eptinezumab) targeting CGRP have been approved for chronic migraine prevention, many patients prefer oral medications because of their ease of use and convenience (6). The first-generation gepants, such as olcegepant and telcagepant, were effective but faced setbacks due to formulation and liver toxicity concerns (7). However, second-generation gepants, particularly atogepant, have demonstrated strong efficacy without significant hepatotoxicity and are now approved for migraine prevention, offering a safer and more reliable option for long-term management (7). Atogepant represents a promising advance as an inaugural oral medication tailored for migraine prevention and is a small-molecule antagonist that targets CGRP receptors. and a half-life of approximately 10 h (8). Preclinical testing has proven its effectiveness, safety, and tolerability in patients with episodic migraines (9). Advanced clinical trials have demonstrated encouraging outcomes, showing substantial reductions in average monthly migraine days (MMDs) and a higher percentage of participants achieving a 50% or greater reduction in MMDs compared to those receiving a placebo (10). Additionally, unlike acute migraine treatments such as NSAIDs and triptans, regular use of this therapy may not pose the risk of medication-overuse headache (MOH) (11).

Existing studies such as ADVANCE and PROGRESS consistently document the efficacy of atogepant for migraine prevention, though some variation exists in study designs and populations, which may influence the generalizability of the findings. To bridge this gap, we thoroughly reviewed and analyzed the existing literature to evaluate the effectiveness and safety of atogepant in treating migraine. Our study included a meticulous literature search, stringent selection criteria, and a detailed study analysis to ensure the credibility and robustness of our analysis.

## Methods

### Search strategy and selection

This study followed the recommendations set forth in the Preferred Reporting Items for Systematic Reviews and Meta-Analyses (PRISMA) guidelines (12). We methodically sourced randomized controlled trials (RCTs) from a range of databases, including the Cochrane Library, PubMed, ScienceDirect, and ClinicalTrials.gov, up to June 21, 2024. Our search strategy encompassed key terms such as “Atogepant,” “migraine,” “migraine disorders,” and “randomized controlled trials,” ensuring a comprehensive approach (Supplementary Table 1). Furthermore, a thorough manual inspection of reference listings from the retrieved papers was conducted to identify recently published studies.

### Data synthesis

All articles identified through the literature search were imported into the EndNote Reference Library (Version X7.5; Clarivate Analytics, Philadelphia, Pennsylvania) to eliminate duplicates and to facilitate the screening process. Two independent reviewers assessed the relevance of articles based on title and abstract, followed by a detailed full-text review according to predefined criteria for inclusion. Any disparities were settled by consensus with a third assessor. Selected studies met the specified criteria for inclusion in the analysis, while exclusion criteria included letters, abstracts, case reports, reviews, and extension studies, reviews.

### Data extraction

Data was extracted from the included studies by two reviewers using Microsoft Excel (Microsoft Corporation, Redmond, WA, United States). This systematic review and meta-analysis focused on the following key outcomes:(I) change in MMDs; (II) change in monthly headache days (MHDs); (III) ≥50% decrease in MMDs; (IV) days of acute medication use; (V) all treatment-emergent adverse events (TEAEs); (VI) treatment-related TEAEs of any kind which included events such as constipation nausea, urinary tract infection and fatigue.; (VII) serious TEAEs such as gastrointestinal symptoms and changes in liver enzymes; (VIII) Role Function-Restrictive domain score of the Migraine-Specific Quality of Life Questionnaire (MSQ); (IX) Performance of Daily Activities domain score of the Activities Impairment in Migraine (AIM) questionnaire; and (X) Physical Impairment domain score of AIM, all of which were assessed at 12 weeks.

### Risk of bias and quality assessment

The Cochrane Risk of Bias Tool for Randomized Controlled Trials (RoB-2) (13) was rigorously applied by two independent reviewers to determine the quality of included RCTs. The evaluation criteria included the randomization process, deviations from intended interventions, incomplete outcome data, outcome assessment, and selection bias. Each study underwent thorough scrutiny to categorize bias risk as “low” or “unclear.”

### Statistical analysis

Statistical analysis was performed using Review Manager software (version 5.4.1; Copenhagen: Published by the Nordic Cochrane Center, The Cochrane Collaboration, 2020). Continuous data were analyzed using Mean Difference (MD), and dichotomous outcomes were assessed using Risk Ratio (RR). Statistical significance was set at *p* < 0.05. Heterogeneity was evaluated using the Higgins I^2^ test (14); and I^2^ value exceeding 50% indicated substantial heterogeneity, prompting a sensitivity analysis. In addition to the pre-specified analyses outlined in our statistical analysis plan, we performed a meta-regression to explore the potential impact of several variables on the effect size of our primary outcome, which is the change in monthly migraine frequency.

## Results

### Study selection and characteristics

**Figure 1:**
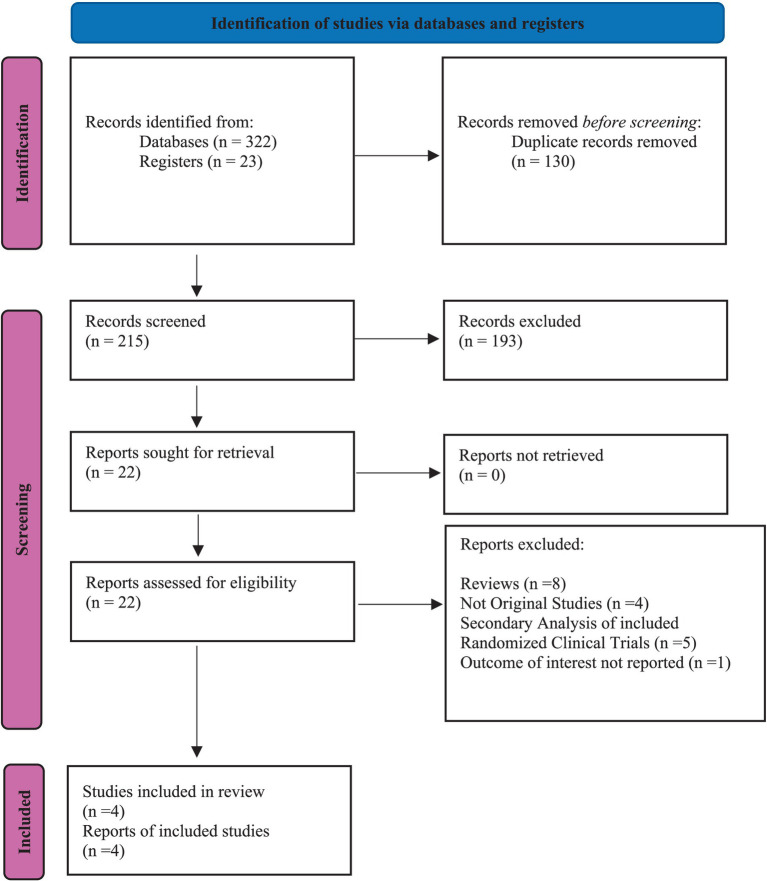
Prisma flow chart.

Our investigation was conducted with utmost care and precision. We meticulously searched indexed databases such as PubMed/MEDLINE, Cochrane Library, Science Direct, and ClinicalTrial.gov, identifying 345 studies that met our search criteria. After eliminating 130 duplicate records, we thoroughly examined the remaining 215 studies for their suitability. Following rigorous evaluation, 193 studies were deemed ineligible, and 22 were selected for further assessment. All 22 reports were subjected to stringent scrutiny to determine eligibility. This thorough and meticulous process, which we believe is crucial for ensuring the reliability and trustworthiness of our findings, is a key part of our study. Ultimately, only 4 studies that matched our strict criteria were chosen for our meta-analysis. The flowchart provided below, in accordance with the PRISMA statement, offers a clear depiction of our methodological screening process (Figure 1). The study characteristics and patients are detailed in Tables 1, 2.

**Table 1:** General characteristics of included studies.

Study	Dose	Total sample	Sample size		Study year	Study design	Country	Follow up duration (Weeks)	Patient category	Primary outcome
			Atogepant	Placebo						
Goadsby et al. 2020	10 mg once daily	825	93	186	2020	Randomized Control Trial	USA	12 weeks	Episodic Migraine	Change from baseline in Monthly Migraine Days
	30 mg once daily		183							
	60 mg once daily		186							
	30 mg twice daily		86							
	60 mg twice daily		91							
Tassoreli et al. 2024	60 mg once daily	313	156	157	2024	Randomized Control Trial	North America/Europe	12 weeks	Episodic Migraine	Change from baseline in Monthly Migraine Days
Ailani et al. 2021	10 mg once daily	902	221	222						
	30 mg once daily		228		2021	Randomized Control Trial	NA	12 weeks	Migraine	Change from baseline in Monthly Migraine Days
	60 mg once daily		231							
Pozo-Rosich et al. 2023	30 mg twice daily	773	257	255	2023	Randomized Control Trial	USA/Canada	12 weeks	Chronic Migraine	Change from baseline in Monthly Migraine Days
	60 mg once daily		261							

**Table 2:** Patient baseline characteristics.

Study	Dose	Mean age		Gender		Weight (kg)		Height		BMI		MMDS baseline		MHDS baseline		Acute medicine use days baseline	
		Atogepant	Placebo	Males	Females	Atogepant	Placebo	Atogepant	Placebo	Atogepant	Placebo	Atogepant	Placebo	Atogepant	Placebo	Atogepant	Placebo
Goadsby et al. 2020	10 mg once daily	39.4 (12.4)	40.5 (11.7)	111	714	NA	NA	79 (8.5)	79 (9.6)	29.9 (7.3)	30.4 (7.6)	7.6 (2.5)	7.8 (2.5)	8.9 (2.7)	9.1 (2.7)	6.2 (3.3)	6.6 (3.2)
	30 mg once daily	41.0 (13.6)								30.0 (7.1)		7.6 (2.4)		8.7 (2.5)		6.6 (3.0)	
	60 mg once daily	40.4 (11.7)								30.0 (7.8)		7.7 (2.6)		8.9 (2.8)		6.8 (3.3)	
	30 mg twice daily	38.5 (11.2)										7.4 (2.4)		8.7 (2.7)		6.2 (3.3)	
	60 mg twice daily	39.7 (11.9)										7.6 (2.6)		8.8 (3.1)		6.4 (3.4)	
Tassoreli et al. 2024	60 mg once daily	40.9 (10.7)	43.4 (10.3)	33	280	71.7 (14.8)	74.0 (16.1)	167.3 (7.9)	167.9 (7.1)	25.6 (4.9)	26.2 (5.2)	9.1 (2.3)	9.3 (2.4)	9.9 (2.4)	10.1 (2.4)	7.5 (2.9)	7.7 (3.3)
Ailani et al. 2021	10 mg once daily	41.4 (12.0)	40.3 (12.8)									7.5 (2.5)	7.5 (2.4)	8.4 (2.8)	8.4 (2.6)	6.6 (3.0)	6.5 (3.1)
	30 mg once daily	42.1 (11.7)		101	801	NA	NA	NA	NA	30.6 (7.8)	30.8 (8.7)	7.9 (2.3)		8.8 (2.6)		6.7 (3.0)	
	60 mg once daily	42.5 (12.4)										7.8 (2.3)		9.0 (2.6)		6.9 (3.2)	
Pozo-Rosich et al. 2023	30 mg twice daily	42.6 (11.9)	42.0 (12.4)	115	663	NA	NA	164.6 (8.3)	164.1 (7.7)	26.2 (6.7)	25.5 (6.0)	18.6 (5.1)	18.9 (4.8)	21.1 (4.1)	21.4 (4.1)	14.5 (7.2)	15.4 (7.0)
	60 mg once daily	41.7 (12.3)						165.0 (8.5)		25.0 (5.5)		19.2 (5.3)		21.5 (4.3)		15.5 (7.4)	

### Risk of bias of included studies

**Figure 2:**
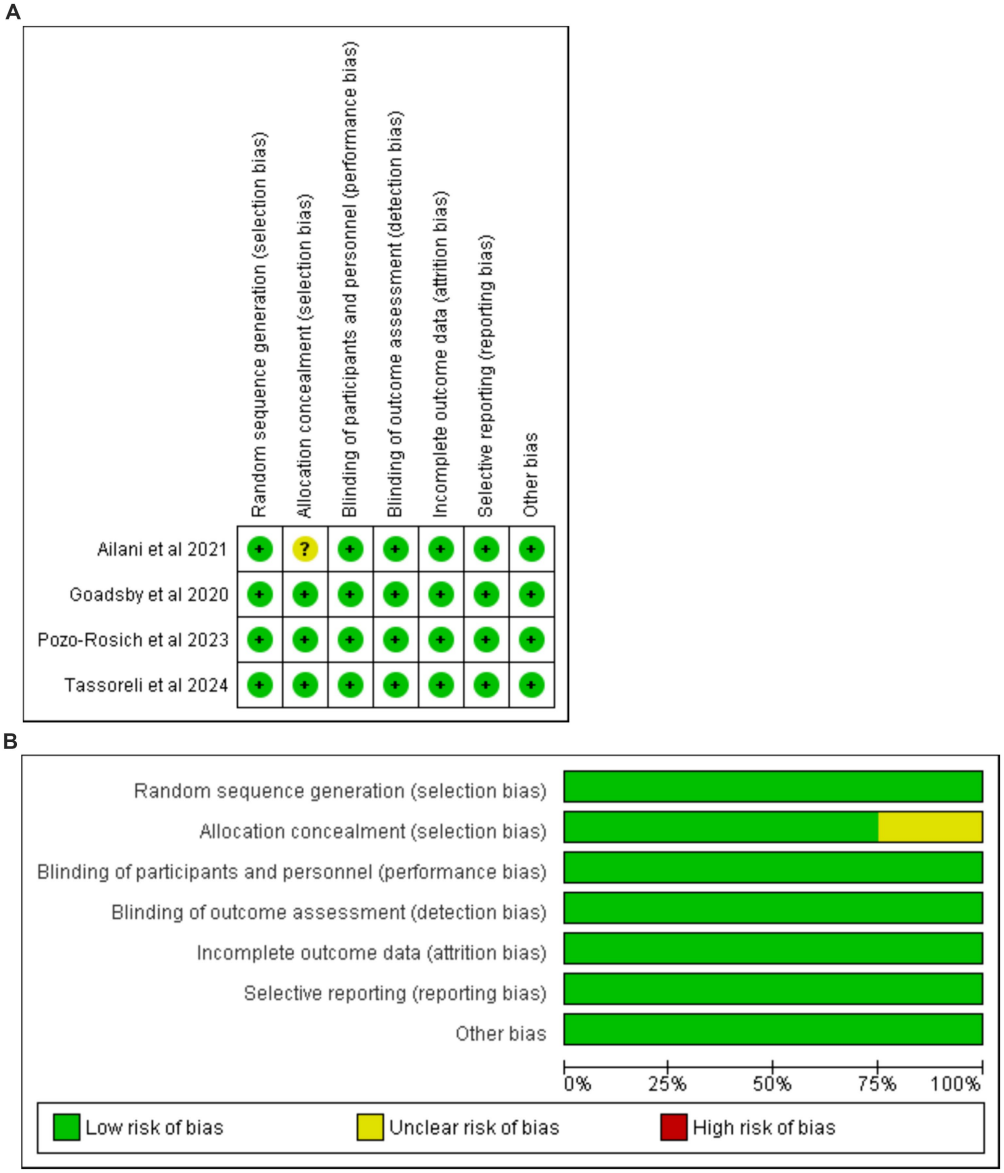
**(A)** Risk of bias graph. **(B)** Risk of bias summary.

The risk of bias was determined in accordance with the Cochrane Handbook for Systematic Reviews and Meta-Analyses. Notably, all reviewed studies were of high quality, as shown in Figures 2A,B (Supplementary Table 2). This high-quality underscore the robustness of our study and is pivotal in our evaluation.

### Primary outcome

#### Change in monthly migraine days

**Figure 3:**
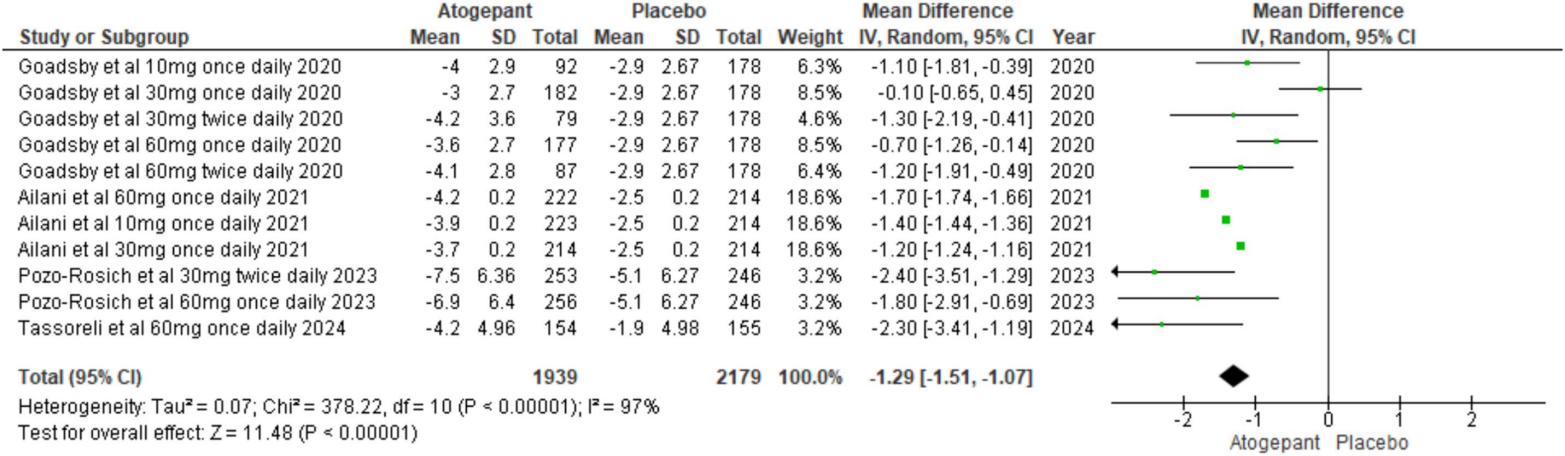
Forest plot of change in monthly migraine days.

The primary focus of these studies was to reduce MMDs over a period of 12 weeks. All four RCTs (13–16) evaluated MMDs as their primary outcome. The findings showed a substantial reduction in migraine days among participants, indicating that the treatment was effective in managing migraine frequency (MD = −1.29, 95% CI [−1.51, −1.07], *p* < 0.00001, I^2^ = 97%). A sensitivity analysis aimed at addressing high heterogeneity did not yield substantial changes (Figure 3).

#### Change in monthly headache days

**Figure 4:**
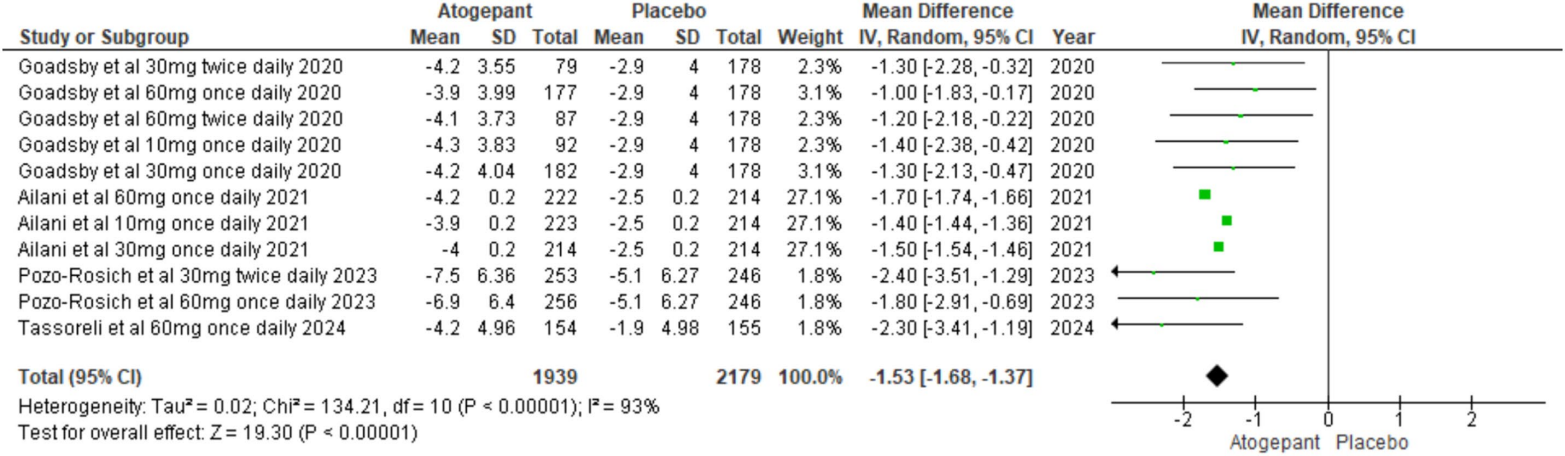
Forest plot of change in monthly headache days.

Several secondary outcomes were assessed across all four RCTs (15–18). Our first secondary outcome analyzed pooled study groups to assess the impact of treatment on monthly headache days (MD = −1.53, 95% CI [−1.68, −1.37], *p* < 0.00001, I^2^ = 93%), indicating a substantial reduction in headache days, supporting the intervention’s efficacy in alleviating headache symptoms (Figure 4). To address the significant heterogeneity, a sensitivity analysis was carried out, identifying that Ailani et al. (16) notably impacted monthly headache day outcomes. Excluding this study, notably reduced I^2^ values (I^2^ = 0%, *p* = 0.43) though it did not substantially alter the overall effect (MD = −1.50, 95% CI [−1.84, −1.16], *p* < 0.00001; Supplementary Figure 1).

#### >50% decrease in monthly migraine days

**Figure 5:**
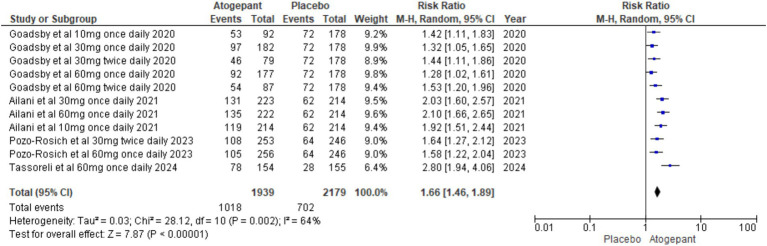
Forest plot of ≥50% reduction in monthly migraine days.

Dichotomous outcomes, specifically the proportion of patients experiencing a > 50% decrease in monthly migraine days, were assessed across all study groups. The overall pooled effect showed a significant benefit of the intervention (RR = 1.66, 95% CI [1.46, 1.89], *p* < 0.00001), with substantial heterogeneity (I^2^ = 64%, *p* = 0.002; Figure 5). To explore the impact of individual studies on this result, a leave-one-out sensitivity analysis was performed, excluding the study by Goadsby et al. (14). This analysis reduced heterogeneity to 41% (*p* = 0.13) and increased the overall effect size to RR = 1.93, 95% CI [1.68, 2.22], *p* < 0.00001 (Supplementary Figure 2).

#### Acute medicine use in days

**Figure 6:**
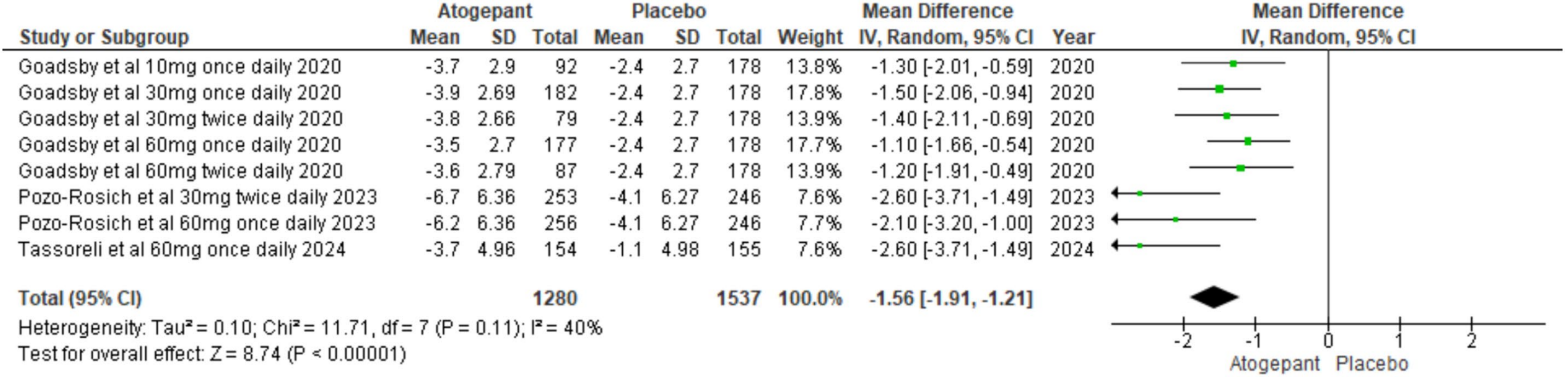
Forest plot of acute medication use days.

All randomized controlled trials (15–18) included in the analysis reported this outcome. The pooled analysis across all study groups indicated a substantial decline in acute medication use days with treatment, in contrast to the control group (MD = −1.56, 95% CI [−1.91, −1.21], *p* < 0.00001, I^2^ = 40%). This finding underscores the effectiveness of treatment in decreasing reliance on acute medications for managing migraine symptoms, as evidenced by the negative MD (Figure 6).

#### Any TEAEs

All RCTs (15–18) reported this outcome. The overall effect of TEAEs was significant (RR = 1.11, 95%CI [1.02, 1.21], *p* = 0.02, I^2^ = 57%) and heterogeneity was significant (*p* = 0.009); thus, a sensitivity analysis was performed for Ailani et al. (16), which resulted in an overall decline of heterogeneity to 0%. The overall representation after sensitivity analysis changed to (RR = 1.20, 95%CI [1.12, 1.28], *p* < 0.00001). This suggests that individuals in the treatment group are more likely to experience any TEAE than those in the control group, indicating a notable relationship between the treatment and the increased risk of experiencing TEAEs compared to the control group before and after sensitivity analysis (Supplementary Figures 3A,B).

#### Any treatment-related TEAEs

All RCTs (15–18) assessed this outcome. The overall effect showed (RR = 1.57, 95%CI [1.36, 1.82], *p* < 0.0001, I^2^ = 7%; Supplementary Figure 4).

#### Serious TEAEs

All RCTs (15–18) reported this outcome, showing no significant change in serious treatment-emergent adverse events across groups (RR = 0.99, 95% CI [0.61, −1.60], *p* = 0.96; I^2^ = 0%; Supplementary Figure 5).

#### Role function-restrictive score on the migraine-specific quality of life questionnaire

Two studies reported this outcome (15, 18). The treatment arm included 1,168 patients in atogepant group and 1,134 in placebo group. Pooled analysis showed (MD = 10.13 95%CI [9.56, 10.71], *p* < 0.0001, I^2^ = 84%). Heterogeneity was significant (*p* < 0.0001). A Leave-one-out analysis was performed by Ailani et al. (16), which reduced heterogeneity to 0%. The overall effect was MD = 7.00, 95%CI [4.26, 9.74], *p* < 0.00001. This suggests that after the exclusion of Ailani et al. (16), heterogeneity was effectively addressed, and the overall effect remained statistically significant, indicating a positive impact of the treatment on the MSQ Function-Restrictive domain at 12 weeks (Supplementary Figures 6A,B).

#### Performance of daily activities score on the AIM-D

The outcomes were discussed by Ailani et al. and Pozo-Rosich et al. (15, 18). Furthermore, the treatment group comprised 1,168 individuals in the atogepant group and 134 participants in the placebo group (MD = −2.81, 95%CI [−3.84, −1.78], *p* < 0.0001 I^2^ = 100%). Leave-one-out was performed because of significant heterogeneity (*p* < 0.0001), resulting in the subsequent removal of Ailani et al. (18), which reduced heterogeneity to 13%, after leaving one-out sensitivity analysis (MD = −4.15, 95%CI [5.62, −2.68]; *p* < 0.00001). After eliminating the trial by Ailani et al. (18), heterogeneity decreased, with a statistically significant improvement in daily activities at 12 weeks (Supplementary Figures 7A,B).

#### Physical impairment score on the AIM-D

Ailani et al. and Pozo-Rosich et al. (15, 18) evaluated this outcome in 1168 atogepant and 1,134 placebo subjects. The pooled analysis indicated (MD = −2.16, 95% CI [−2.88, −1.43], *p* < 0.00001, I^2^ = 99%). Without Ailani et al. 2021 (18), the impact was (MD = -3.45, 95% CI [−4.92, −1.98], *p* < 0.00001, I^2^ = 13%). At 12 weeks, there was a substantial improvement in the AIM-D Physical Impairment domain with less heterogeneity, highlighting the effectiveness of the treatment (Supplementary Figures 8A,B).

### Subgroup analysis

#### Change in monthly migraine days

No clear dose–response relationship was observed across the different dosages. For the 10 mg once daily dosage, the mean difference (MD) was −1.40 (95% CI [−1.44, −1.36], *p* < 0.00001). The 30 mg once daily dosage showed an MD of-0.69 (95% CI [−1.76, 0.39], *p* = 0.21, I^2^ = 93%). For the 60 mg once daily dosage, the MD was −1.53 (95% CI [−2.17, −0.90], *p* < 0.00001, I^2^ = 78%). The 30 mg twice daily dosage resulted in an MD of-1.80 (95% CI [−2.87, −0.72], *p* = 0.001, I^2^ = 57%), and the 60 mg twice daily dosage had an MD of-1.20 (95% CI [−1.91, −0.49], *p* = 0.0009; Supplementary Figure 9A).

#### Change in monthly headache days

A similar trend of no dose–response relationship was observed across different dosages. For the 10 mg once daily dosage, the mean difference (MD) was −1.40 (95% CI [−1.44, −1.36]; *p* < 0.00001). For the 30 mg once daily dosage, the MD was −1.50 (95% CI [−1.54, −1.46]; *p* < 0.00001), with an I^2^ of 27%, indicating low heterogeneity. The 60 mg once daily dosage showed an MD of −1.67 (95% CI [−1.99, −1.34]; *p* < 0.00001), with an I^2^ of 23%, also reflecting low heterogeneity. For the 30 mg twice daily dosage, the MD was −1.82 (95% CI [−2.89, −0.74]; *p* < 0.0009), with a higher I^2^ of 53%, suggesting moderate heterogeneity. Finally, for the 60 mg twice daily dosage, the MD was −1.20 (95% CI [−2.18, −0.22]; *p* = 0.02), but the results showed a broader confidence interval with no significant heterogeneity reported (Supplementary Figure 9B).

#### ≥50% decrease in monthly migraine days

The percentage of participants achieving ≥50% reduction increased with the dose. There was a lack of a consistent dose–response effect between all subgroups: 10 mg once daily (RR = 1.66 [95% CI 1.23, 2.23]; *p* = 0.0008), 30 mg once daily (RR = 1.63 [95% CI 1.07, 2.49]; *p* = 0.02; I^2^ = 85%), 60 mg once daily (RR 1.82 [95% CI 1.34, 2.48]; *p* = 0.0001; I^2^ = 82%), 30 mg twice daily (RR: 1.54 [95% CI: 1.28, 1.84]; *p* < 0.00001; I^2^ = 0%), and 60 mg twice daily (RR 1.53 [95% CI 1.20, 1.96]; *p* = 0.0005; Supplementary Figure 9C).

#### Acute medication use in days

Reduction in acute medication use also showed dose dependency. For the 10 mg once daily dosage, the mean difference (MD) was −1.30 (95% CI [−2.01, −0.59]; *p* = 0.0004). For the 30 mg once daily dosage, the MD was −1.50 (95% CI [−2.06, −0.94]; *p* < 0.00001). For the 60 mg once daily dosage, the MD was −1.84 (95% CI [−2.81, −0.86]; *p* = 0.0002; I^2^ = 71%). For the 30 mg twice daily dosage, the MD was −1.92 (95% CI [−3.09, −0.76]; *p* = 0.001; I^2^ = 69%). For the 60 mg twice daily dosage, the MD was −1.20 (95% CI [−1.91, −0.49]; *p* = 0.0009). These results demonstrate that higher doses are associated with a substantial reduction in acute medication use (Supplementary Figure 9D).

#### Any TEAEs

There was no clear dose–response relationship observed across the subgroups. For the 10 mg once daily dosage, the relative risk (RR) was 1.11 (95% CI [0.78, 1.56]; *p* = 0.57; I^2^ = 85%). For the 30 mg once daily dosage, the RR was 1.08 (95% CI [0.79, 1.48]; *p* = 0.64; I^2^ = 85%). For the 60 mg once daily dosage, the RR was 1.09 (95% CI [0.93, 1.26]; *p* = 0.28; I^2^ = 65%). For the 30 mg twice daily dosage, the RR was 1.17 (95% CI [1.02, 1.34]; *p* = 0.02; I^2^ = 0%). For the 60 mg twice daily dosage, the RR was 1.18 (95% CI [0.94, 1.48]; *p* = 0.16). These results indicate variability in the incidence of treatment-emergent adverse events (TEAEs; Supplementary Figure 9E).

#### Any treatment related TEAEs

Treatment-related treatment-emergent adverse events (TEAEs) also showed no clear dose–response relationship. For the 10 mg once daily dosage, the relative risk (RR) was 1.72 (95% CI [0.77, 3.83]; *p* = 0.18; I^2^ = 80%). For the 30 mg once daily dosage, the RR was 1.45 (95% CI [1.04, 2.02]; *p* = 0.03; I^2^ = 0%). For the 60 mg once daily dosage, the RR was 1.64 (95% CI [1.25, 2.15]; *p* = 0.0004; I^2^ = 26%). For the 30 mg twice daily dosage, the RR was 1.43 (95% CI [1.05, 1.97]; *p* = 0.03; I^2^ = 0%). For the 60 mg twice daily dosage, the RR was 1.64 (95% CI [1.02, 2.63]; *p* = 0.04; Supplementary Figure 9F).

#### Serious TEAEs

No clear association was observed between higher dosages and more serious treatment-emergent adverse events (TEAEs). The dose–response differences between the subgroups were as follows: 10 mg once daily (RR 1.00, 95% CI [0.22, 4.54]; *p* = 1.00; I^2^ = 0%), 30 mg once daily (RR 0.63, 95% CI [0.12, 3.23]; *p* = 0.58; I^2^ = 0%), 60 mg once daily (RR 0.98, 95% CI [0.39, 2.48]; *p* = 0.97; I^2^ = 13%), 30 mg twice daily (RR 1.20, 95% CI [0.55, 2.61]; *p* = 0.65; I^2^ = 0%), and 60 mg twice daily (RR 0.41, 95% CI [0.02, 8.38]; *p* = 0.56). These results indicate that there was no significant increase in serious TEAEs across the different dosages (Supplementary Figure 9G).

#### Role function-restrictive score on the migraine-specific quality of life questionnaire

No dose–response improvement was observed. The results were as follows: 10 mg once daily (MD 9.90, 95% CI [9.60, 10.20]; *p* < 0.00001), 30 mg once daily (MD 10.10, 95% CI [9.80, 10.40]; *p* < 0.00001), 60 mg once daily (MD 8.86, 95% CI [4.33, 13.40]; *p* = 0.0001; I^2^ = 82%), and 30 mg twice daily (MD 7.90, 95% CI [4.03, 11.77]; *p* < 0.00001; Supplementary Figure 9H).

#### Performance of daily activities score on the AIM-D

Dose–response improvement was observed, 10 mg once daily (MD = −1.20, 95% CI [−1.29, −1.11]; *p* < 0.00001), 30 mg once daily (MD = −2.50, 95% CI [−2.59, −2.41], *p* < 0.00001), 60 mg once daily (MD-3.30, 95% CI [−3.39, −3.21], *p* < 0.00001, I^2^ = 0%), and 30 mg twice daily (MD = −4.90, 95% CI [−6.84, −2.96], *p* < 0.00001). These results suggested that higher doses resulted in greater improvements in the performance of daily activities in the AIM-D domain (Supplementary Figure 9I).

#### Physical impairment score on the AIM-D

Dose–response improvement observed, 10 mg once daily (MD = −1.10, 95% CI [−1.18, −1.02], *p* < 0.00001), 30 mg once daily (MD = −2.00, 95% CI [−2.08, −1.92], *p* < 0.00001), 60 mg once daily (MD = −2.50, 95% CI −[2.58, −2.42] *p* < 0.00001, I^2^ = 0%), and 30 mg twice daily (MD = −4.20, 95% CI [−6.15, −2.25], *p* < 0.00001). These results suggested that higher doses resulted in greater improvements in the physical impairment domain of AIM-D (Supplementary Figure 9J).

### Meta-regression

We evaluated the potential impact of mean age, body mass index (BMI), proportion of male participants, and migraine duration on the effect size of our primary outcome, which was the change in monthly migraine. The findings were as follows: mean age (Coeff: −0.4448, *p* = 0.0185), male sex percentage (Coeff: 0.1053, *p* = 0.1535), BMI (Coeff: 0.1951, *p* = 0.0280), and duration of migraine (Coeff: −0.0142, *p* = 0.9838; Supplementary Figures 10A–D).

## Discussion

Our meta-analysis, a unique and comprehensive study of four randomized controlled trials (RCTs) involving 2,713 patients, stands out in the existing body of research on CGRP antagonists (15–18). It examined the efficacy of atogepant in reducing monthly migraine days (MMDs) and headache days compared to placebo. The primary outcome, the change in MMDs over 12 weeks, showed a significant reduction with atogepant at doses of 10 mg, 30 mg, and 60 mg. Secondary outcomes, such as changes in monthly headache days, the percentage of participants experiencing a ≥ 50% decrease in MMDs, days using acute medication, and several patient-reported outcomes, consistently indicated that atogepant outperformed placebo. These findings highlight the effectiveness of atogepant in reducing migraine burden.

A previous meta-analysis by Lattanzi et al. (19) evaluated atogepant for episodic migraine prevention, based on two trials (16, 18). Their findings demonstrated substantial reductions in monthly migraine days with atogepant doses of 10 mg, 30 mg, and 60 mg compared to placebo. While side effects and treatment cessation rates were comparable between groups, atogepant was associated with increased incidences of constipation and nausea. The study highlighted atogepant’s efficacy and tolerability in preventing episodic migraines in adults. Our meta-analysis builds on these findings by including both episodic and chronic migraine patients. Documenting chronic migraine is particularly important because this condition often presents with a more severe and persistent disease course, leading to greater functional impairment and decreased quality of life compared to episodic migraine. Chronic migraine patients frequently experience more significant treatment challenges and have different therapeutic needs. By evaluating atogepant in this broader patient population, our study provides a more comprehensive understanding of its efficacy and safety across diverse migraine types. We also examine secondary outcomes, such as role function, daily activities, and physical impairment, which are critical for assessing the overall impact on patients’ quality of life.

Current guidelines for migraine treatment, such as those from the American Headache Society (AHS) (19), European Federation of Neurological Societies (EFNS) (20), emphasize evidence-based approaches to both acute and preventive therapies. These guidelines recommend a range of treatments, including nonsteroidal anti-inflammatory drugs (NSAIDs), triptans, beta-blockers, antiepileptic drugs, and monoclonal antibodies that target the CGRP receptors. The 2024 NICE guidelines advise considering atogepant for migraine prevention in adults with a minimum of four migraine days monthly, contingent upon inadequate response to at least three other preventive medications (21). Atogepants have received FDA approval in September 2021, and their emergence as oral CGRP receptor antagonists has sparked interest in clinical practice and ongoing research. Future updates to guidelines are anticipated as more data becomes available regarding its efficacy, safety profile, and role in migraine management strategies. Atogepant is a selective CGRP receptor antagonist, blocking CGRP-R1, thereby inhibiting its vasodilatory and pro-inflammatory effects by preventing CGRP binding to receptor activity-modifying protein 1 (RAMP1) and CGRP-R1 complex formation (22, 24). Atogepant’s specificity for CGRP-R1 selectively targets migraine pathophysiology, avoiding broader systemic effects associated with non-selective CGRP interventions, thereby effectively mitigating migraine symptoms (23, 25). Although, monoclonal antibodies can promote adherence through monthly or quarterly injections, atogepant provides a convenient oral alternative for patients who either prefer non-injectable treatments or cannot self-administer injections (22). Its pharmacokinetic profile supports once-daily dosing, ensuring sustained CGRP receptor antagonism that effectively reduces migraine frequency and severity by chronically modulating neuronal excitability and inflammatory responses, distinct from acute symptom-targeting treatments (22). The previous meta-analysis by Simona Lattanzi did not explore the dose–response relation of atogepant, whereas our meta-analysis addresses both dose and frequency of the medication (19). Our subgroup analysis revealed no clear dose–response pattern for reducing monthly migraine or headache days, though all doses demonstrated efficacy. However, higher heterogeneity, particularly with the 30 mg and 60 mg once-daily doses, complicates the interpretation of these results. In contrast, higher doses, such as 60 mg once daily, were associated with greater improvements in functional outcomes, including daily activity performance and physical impairment, indicating a stronger dose–response effect on quality of life. Additionally, these higher doses reduced the need for acute medication, though this benefit was not linked to increased dosing frequency. Our analysis also examined the effect of dosing frequency, cocluding that twice-daily regimens did not consistently offer additional benefits over once-daily dosing in reducing migraines. While the 30 mg and 60 mg twice-daily regimens showed some improvement in outcomes like reduced medication use and functional impairment, the effect was not significantly superior to once-daily dosing, indicating limited added value from more frequent dosing for overall migraine control. These insights emphasize the importance of personalized treatment, where lower doses can be effective and preferable for some patients, while higher doses may be reserved for those with more severe symptoms. Furthermore, the meta-analysis also integrates findings from the PROGRESS (15) and ELEVATE (17) trials, demonstrating atogepant’s efficacy in both chronic and episodic migraine settings. PROGRESS affirmed significant reductions in MMDs with atogepant compared to placebo, comparable to injectable therapies, while ELEVATE underscored its efficacy in treatment-resistant episodic migraine cases. These findings collectively underscore atogepant’s role as a robust, flexible, and potentially first-line therapy in migraine management. While atogepant effectively reduces migraine frequency, the meta-analysis highlighted important safety considerations. In our study, patients treated with atogepant experienced higher rates of treatment-emergent adverse events (TEAEs), such as constipation nausea, urinary tract infection, fatigue. Serious TEAEs were rare and occurred similarly in both groups, suggesting an overall manageable safety profile. While serious TEAEs were rare and occurred similarly in both groups, indicating an overall manageable safety profile, the increased incidence of these common TEAEs highlights the need for clinicians to carefully balance the benefits of migraine reduction with the risks of potential side effects.

Our meta-analysis, with its numerous noteworthy strengths, provides a comprehensive and reliable assessment of atogepant’s efficacy and safety. Firstly, it includes a large sample size derived from multiple randomized controlled trials (RCTs), which strengthens the statistical robustness and applicability of our findings. Secondly, the analysis comprehensively examines various outcomes, including reductions in monthly migraine days, changes in headache days, and patient-reported outcomes, providing a holistic view of atogepant’s efficacy. Third, by examining multiple doses of atogepant (10 mg, 30 mg, and 60 mg), the meta-analysis investigates dose–response relationships, which are critical for improving treatment regimens. Our investigation will significantly impact the establishment of atogepant’s safety profile. Our regression analysis revealed that younger patients and those with lower BMI experienced greater reductions in monthly migraine days, with atogepant likely due to less complex migraine pathophysiology or more favorable drug metabolism. In contrast, the proportion of male participants and the duration of migraine did not significantly affect treatment outcomes, suggesting that gender and migraine chronicity have minimal impact on atogepant’s efficacy, offereing novel insights into drugs efficacy. Despite its strengths, this study faces several limitations. Firstly, significant heterogeneity was observed across the included trials; thereby, a leave-one-out analysis was carried out, which found out removing the Ailani et al. (18) study significantly reduced heterogeneity, suggesting that unique factors in this study, such as its dosing regimen, exclusion criteria, and geographical diversity, contributed to the observed variability. Additionally, differences in statistical methods, data handling, and endpoint definitions may have further influenced the outcomes, necessitating sensitivity analyses to mitigate its impact, which may affect the robustness of the findings. Secondly, this study primarily involved female patients and was conducted in Western settings. This demographic skew highlights the need for cautious monitoring and may limit the generalizability of the results across diverse patient groups and other regions or settings. Exploring other geographic regions and healthcare contexts is recommended for future trials to broaden the scope of existing literature. Thirdly, all the included trials in the study primarily focused on a 12-week treatment period, providing limited information on the sustained safety and effectiveness of atogepant, which is crucial for chronic migraine management. Lastly, While the leave-one-out analysis revealed that removing the Ailani et al. study reduced heterogeneity, we acknowledge that this study did not differ significantly from the others in terms of overall effect sizes.

## Conclusion

In conclusion, our meta-analysis confirms atogepant as an effective option for migraine prevention, reducing migraine frequency in both episodic and chronic cases. Higher doses improve daily functioning, though once-daily dosing generally suffices for most patients. The study highlights the importance of personalized treatment to balance efficacy with side effects like constipation and nausea. While atogepant offers flexibility and convenience, longer-term studies are needed to further assess its sustained safety and efficacy across diverse populations.

## Data Availability

The original contributions presented in the study are included in the article/Supplementary material, further inquiries can be directed to the corresponding author.
