# Corrigendum: Atogepant for migraine prevention: a meta-analysis of safety and efficacy in adults

**DOI:** 10.3389/fneur.2025.1577914

**Published:** 2025-04-10

**Authors:** Adarsh Raja, Rabia Asim, Muhammad Hamza Shuja, Sandesh Raja, Tazheen Saleh Muhammad, Simran Bajaj, Abdul Hadi Ansari, Hamza Ali, Iffat Ambreen Magsi, Muhammad Hammad Faridi, Hamza Ali Hasnain Sheikh, Muhammad Junaid Imran, Muhammad Ahmed, Muhammad Sohaib Asghar

**Affiliations:** ^1^Department of Internal Medicine, Shaheed Mohtarma Benazir Bhutto Medical College Lyari, Karachi, Pakistan; ^2^Department of Internal Medicine, Dow University of Health Sciences, Karachi, Pakistan; ^3^Department of Internal Medicine, Shaheed Mohtarma Benazir Bhutto University, Larkana, Pakistan; ^4^Department of Internal Medicine, AdventHealth, Sebring, FL, United States; ^5^Department of Internal Medicine, Mayo Clinic, Rochester, MN, United States

**Keywords:** atogepant, CGRP, migraine, headache, meta-analysis

In the published article, there was an error. Two reviewer comments inadvertently appeared in the final published article. Two sentences have been removed from the **Results section**, *Secondary outcomes*. These sentences previously stated:

Regarding change in monthly headache days, I do not see the relevance to do a sensitivity analysis. Also after excluding Ailani et al., the mean value with 95% CI does not appear so different than the previous one.

A sentence has been removed from the **Results section**, *Meta-regression, first paragraph*. This sentence previously stated:

The meta-regression is not anticipated in the statistical analysis plan. What is the purpose of such analysis?

The authors apologize for this error and state that this does not change the scientific conclusions of the article in any way. The original article has been updated.

