# Supplementary material for: Atogepant for migraine prevention: a meta-analysis of safety and efficacy in adults
Source: Front Neurol. 2024 Sep 27;15:1468961. doi: 10.3389/fneur.2024.1468961 (PMC11466836; doi:10.3389/fneur.2024.1468961)
Supplement: Supplementary file 1 [file Data_Sheet_1.docx]

**Supplementary Table 1.** Search Strategies for different Databases

| **Database** | **Search String** |
| --- | --- |
| PubMed/MEDLINE  (97 Results) | ("atogepant"[Supplementary Concept] OR "atogepant"[All Fields]) AND ("migrain"[All Fields] OR "migraine disorders"[MeSH Terms] OR ("migraine"[All Fields] AND "disorders"[All Fields]) OR "migraine disorders"[All Fields] OR "migraine"[All Fields] OR "migraines"[All Fields] OR "migraine s"[All Fields] OR "migraineous"[All Fields] OR "migrainers"[All Fields] OR "migrainous"[All Fields]) |
| Cochrane Library  (127 Results) | atogepant AND migraine |
| Science Direct  (98 Results) | atogepant AND migraine |
| Clinical Trial Govt  (23 results) | Intervention: atogepant; Condition: migraine |

**Supplementary Table 2:** Risk of Bias assessment

|  | Cochrane Risk-of-Bias Tool | | |  |
| --- | --- | --- | --- | --- |
|  | Bias | Risk of bias | Author judgment | |
| \| Goadsbay et al. 2020 \| \| --- \| \|  \| \|  \| \|  \| \|  \| | Random sequence generation (selection bias) | Low Risk | An automated interactive web-based response system was used for randomization. | |
|  | Allocation concealment (selection bias) | Low Risk | Study participants, site personnel, and all study sponsor personnel were masked to treatment allocations. | |
|  | Blinding of participants and personnel (performance bias) | Low Risk | Identical blister cards and over-encapsulation were implemented to maintain masking. | |
|  | Blinding of outcome assessment (detection bias) | Low Risk | Outcome assessors were blinded to intervention allocation, reducing the risk of detection bias. Furthermore, participants used electronic diaries. | |
|  | Incomplete outcome data (attrition bias) | Low Risk | No incomplete outcome data was reported, minimizing the risk of attrition bias. | |
|  | Selective reporting (reporting bias) | Low Risk | There is no evidence of selective outcome reporting, reducing the risk of reporting bias. | |
|  | Other bias | Low Risk | No other biases were identified in the study that could significantly impact the results. | |
| Tassorelli et al. 2024 | Random sequence generation (selection bias) | Low Risk | The study implemented a randomization cap to ensure a balanced distribution of participants with specific baseline characteristics. | |
|  | Allocation concealment (selection bias) | Low Risk | Although the allocation concealment process was not explicitly described, the trial was double-blind, and patients and personnel were unaware of individual patient identification and treatment assignments. Therefore, it's likely that allocation concealment was effectively implemented, minimizing biased allocation to interventions. | |
|  | Blinding of participants and personnel (performance bias) | Low Risk | Participants, investigators, and study site personnel were rigorously blinded to treatment assignment throughout the trial. | |
|  | Blinding of outcome assessment (detection bias) | Low Risk | Outcome assessors were blinded to intervention allocation, decreasing the risk of detection bias. The blinding of participants and personnel extended to outcome assessment, with patient-reported outcomes recorded using electronic diaries and tablets. | |
|  | Incomplete outcome data (attrition bias) | Low Risk | Incomplete outcome data were handled appropriately, minimizing the risk of attrition bias. The study specified the populations excluded from certain analyses and the rationale behind these exclusions. | |
|  | Selective reporting (reporting bias) | Low Risk | There is no evidence of selective outcome reporting, reducing the risk of reporting bias. | |
|  | Other bias | Low Risk | No other biases were identified in the study that could significantly impact the results. | |
| Ailani et al. 2021 | Random sequence generation (selection bias) | Low Risk | The study implemented a randomization cap to ensure a balanced distribution of participants with specific baseline characteristics. | |
|  | Allocation concealment (selection bias) | Unclear risk | The allocation concealment process was not explicitly described. | |
|  | Blinding of participants and personnel (performance bias) | Low Risk | The study clearly stated that participants, site personnel, and trial sponsor personnel were unaware of the trial-group assignments, indicating the successful blinding of participants and personnel. | |
|  | Blinding of outcome assessment (detection bias) | Low Risk | Outcome assessors were blinded to intervention allocation, reducing the risk of detection bias. | |
|  | Incomplete outcome data (attrition bias) | Low Risk | Incomplete outcome data were handled appropriately, minimizing the risk of attrition bias. | |
|  | Selective reporting (reporting bias) | Low Risk | There is no evidence of selective outcome reporting, reducing the risk of reporting bias. | |
|  | Other bias | Low Risk | No other biases were identified in the study that could significantly impact the results. | |
| Pozo-Rosich et al 2023 | Random sequence generation (selection bias) | Low Risk | Participants, investigators, and study site personnel were masked to treatment assignment. | |
|  | Allocation concealment (selection bias) | Low Risk | Although the allocation concealment process was not explicitly described, the trial was double-blind, and patients and personnel were unaware of individual patient identification and treatment assignments. Therefore, it's likely that allocation concealment was effectively implemented, minimizing biased allocation to interventions. | |
|  | Blinding of participants and personnel (performance bias) | Low Risk | At the screening (baseline) visit, participants were provided with an eDiary, training, and instructions. | |
|  | Blinding of outcome assessment (detection bias) | Low Risk | Outcome assessors were blinded to intervention allocation, decreasing the risk of detection bias. | |
|  | Incomplete outcome data (attrition bias) | Low Risk | Incomplete outcome data were handled appropriately, minimizing the risk of attrition bias. | |
|  | Selective reporting (reporting bias) | Low Risk | There is no evidence of selective outcome reporting, reducing the risk of reporting bias. | |
|  | Other bias | Low Risk | No other biases were identified in the study that could significantly impact the results. | |

**Supplementary Figure 1:** Leave one out analysis by **Ailani et al. 2021**

**
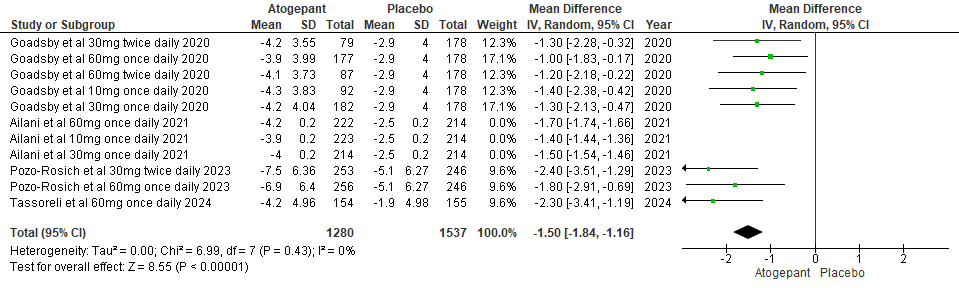
**

**Supplementary Figure 2:** Leave one out analysis by **Goadsby et al. 2020**

**
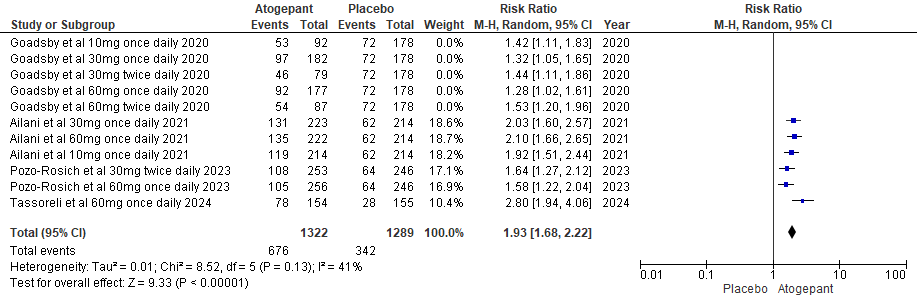
**

**Supplementary Figure 3A:** Any TEAEs

**
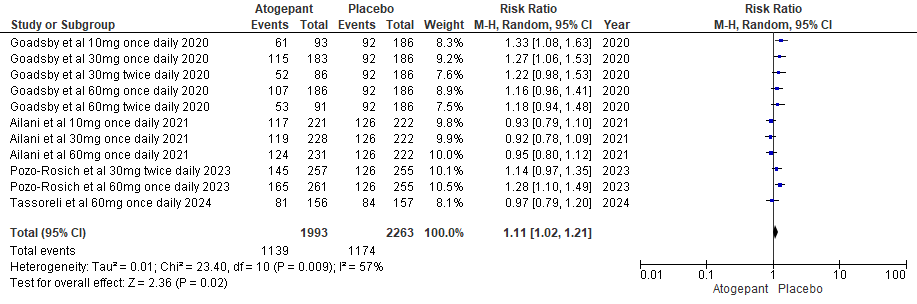
**

**Supplementary Figure 3B:** Leave one out analysis by **Ailani et al. 2021**

**
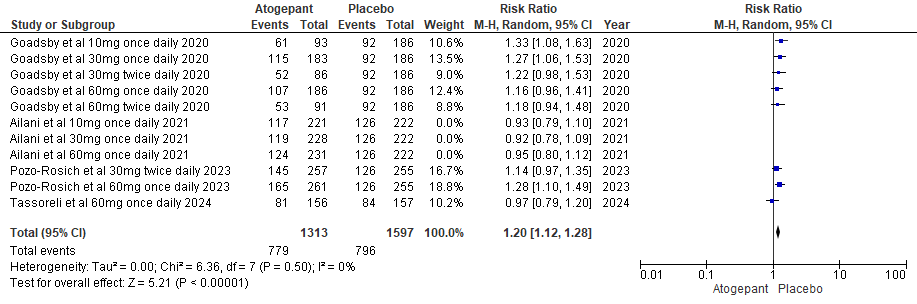
**

**Supplementary Figure 4:** Any treatment-related TEAEs

**
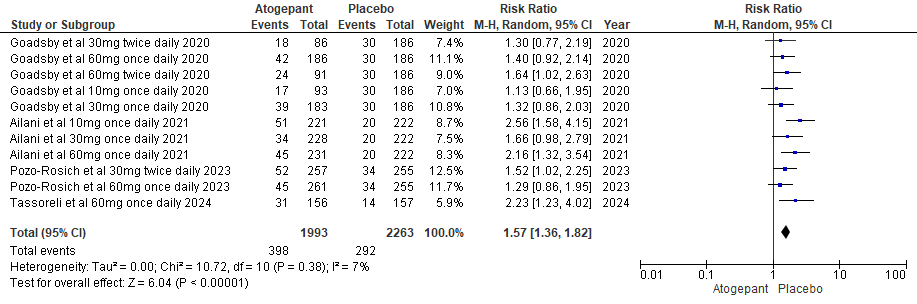
**

**Supplementary Figure 5:** Serious TEAEs

**
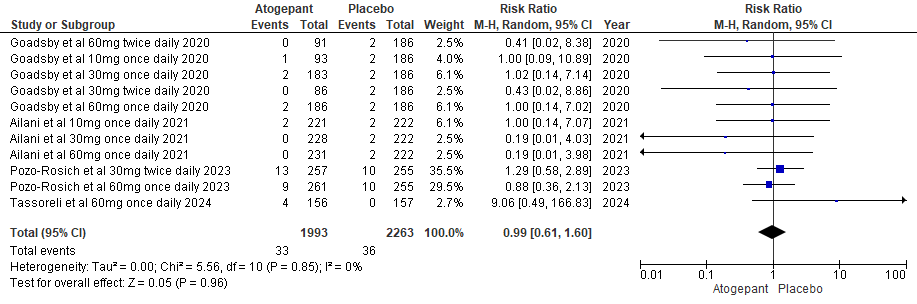
**

**Supplementary Figure 6A:** Score on Role Function–Restrictive domain of MSQ

**
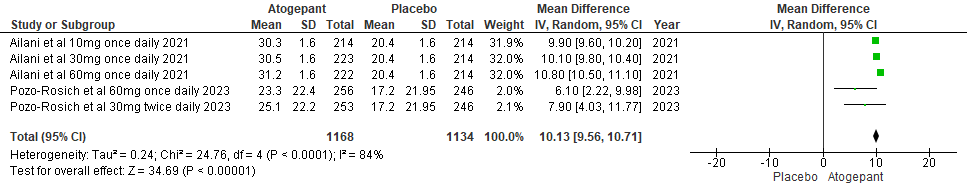
**

**Supplementary Figure 6B:** Leave one out analysis by **Ailani et al. 2021**

**
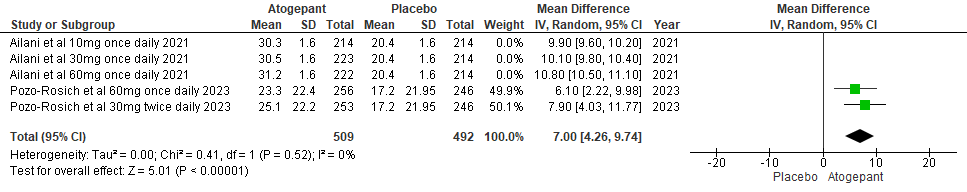
**

**Supplementary Figure 7A:** Score on Performance of Daily Activities domain

**
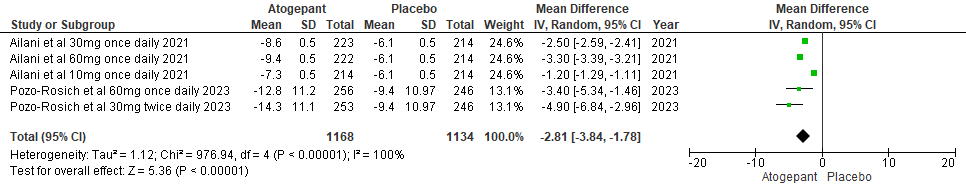
**

**Supplementary Figure 7B:** Leave one out analysis by **Ailani et al. 2021**

**
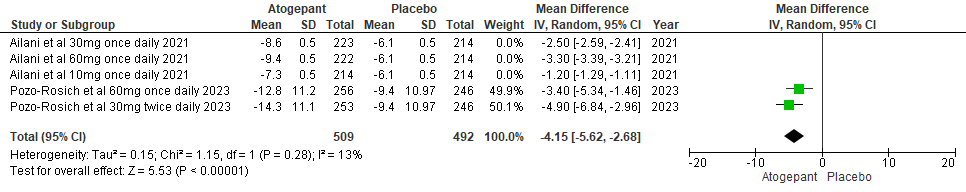
**

**Supplementary Figure 8A:** Score on Physical Impairment domain of AIM-D

**
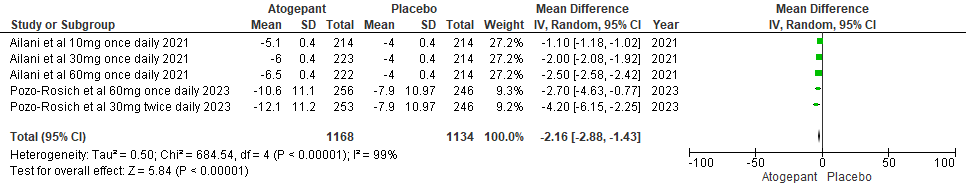
**

**Supplementary Figure 8B:** Leave one out analysis by **Ailani et al. 2021**

**
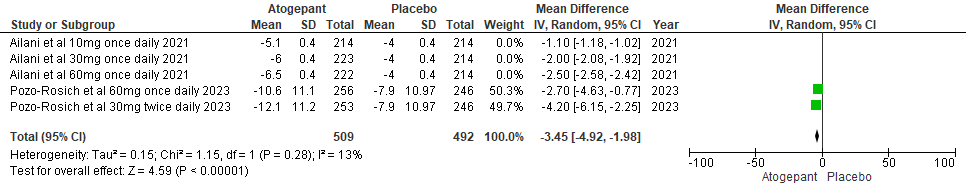
**

**Supplementary Figure 9A:** Change from baseline in Monthly Migraine Days at 12 weeks

**
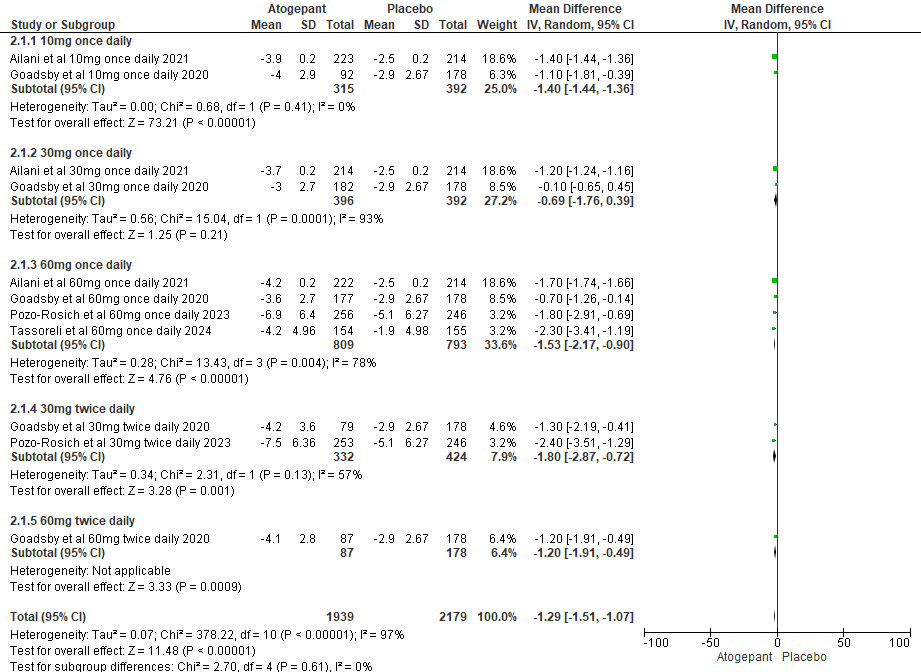
**

**Supplementary Figure 9B:** Change from baseline in Monthly Headache Days at 12 weeks

**
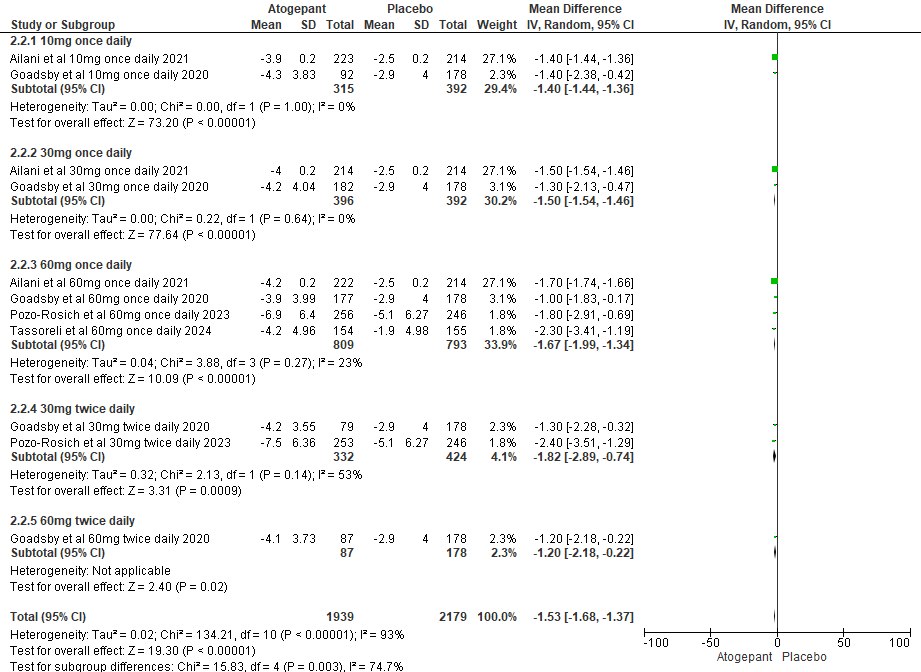
**

**Supplementary Figure 9C:** ≥50% Reduction in Monthly Migraine Days

**
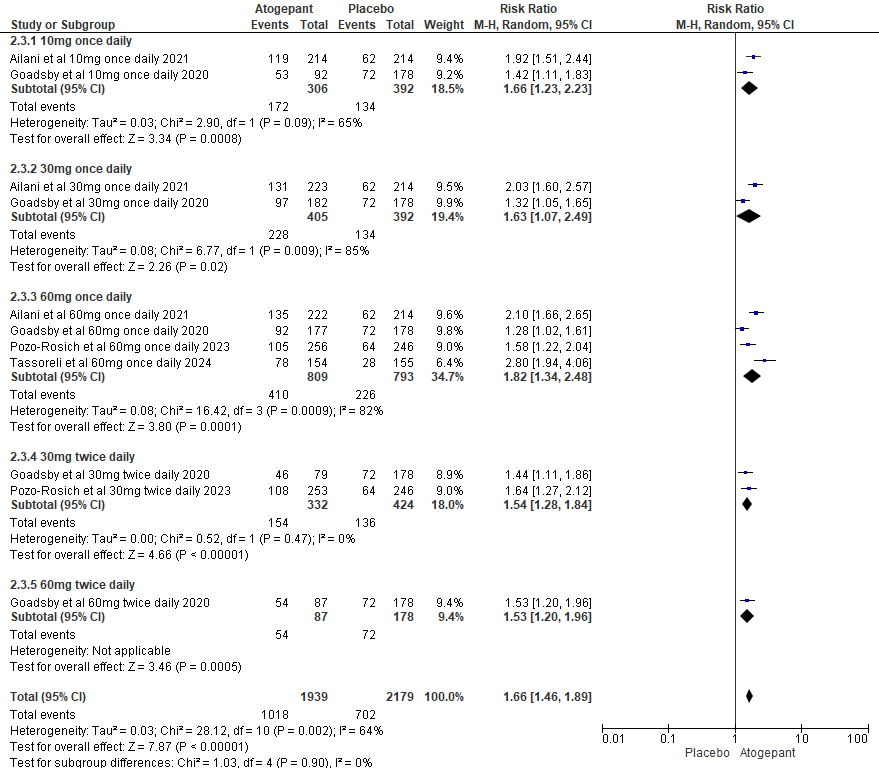
**

**Supplementary Figure 9D:** Acute Medication Use Days at 12 weeks

**
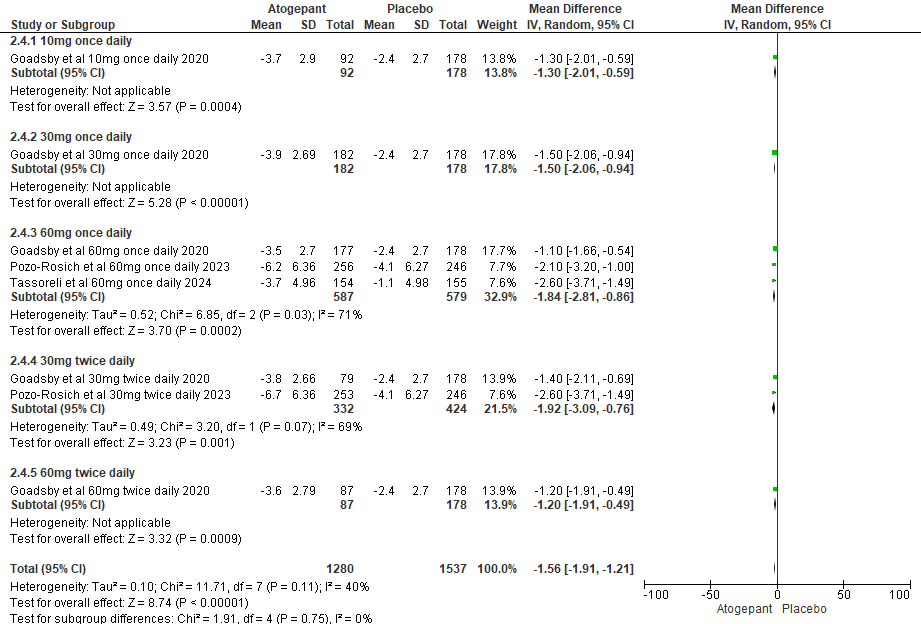
**

**Supplementary Figure 9E:** Any TEAEs

**
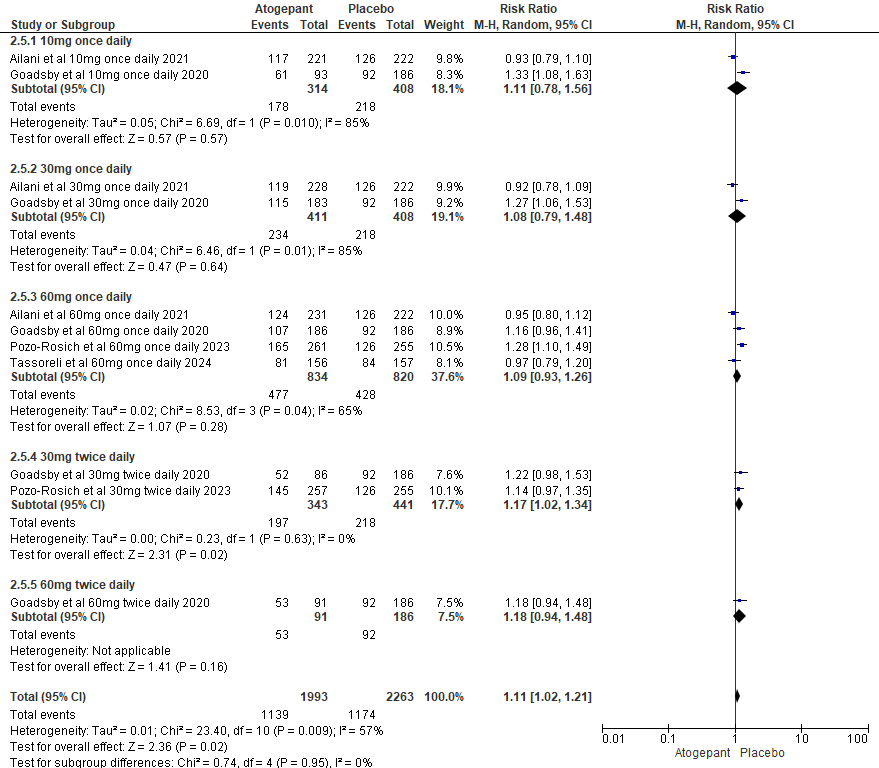
**

**Supplementary Figure 9F:** Any treatment-related TEAEs

**
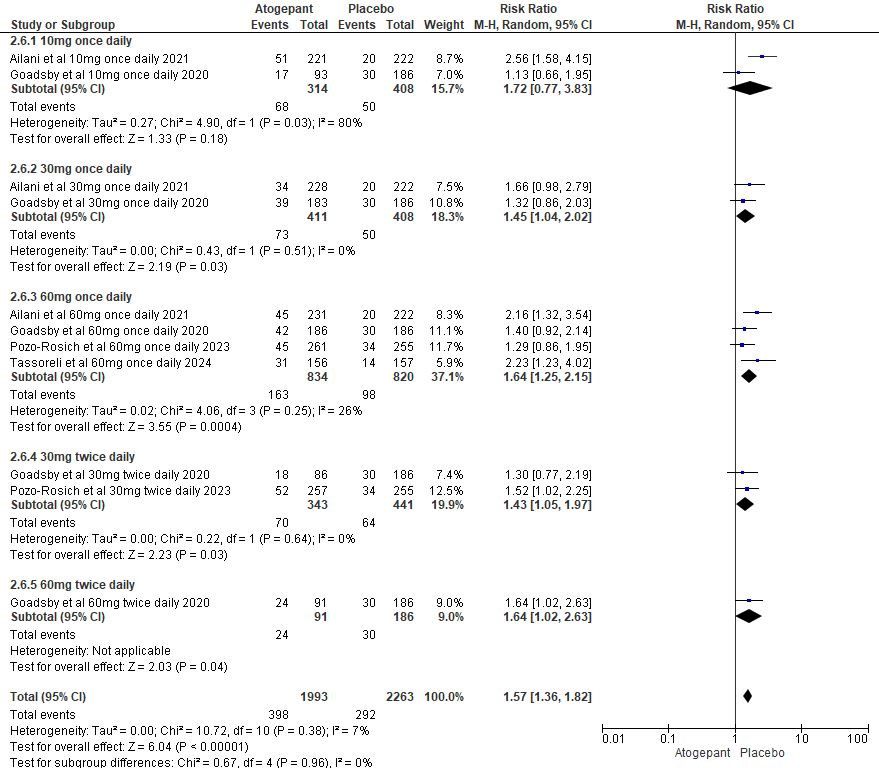
**

**Supplementary Figure 9G:** Serious TEAEs

**
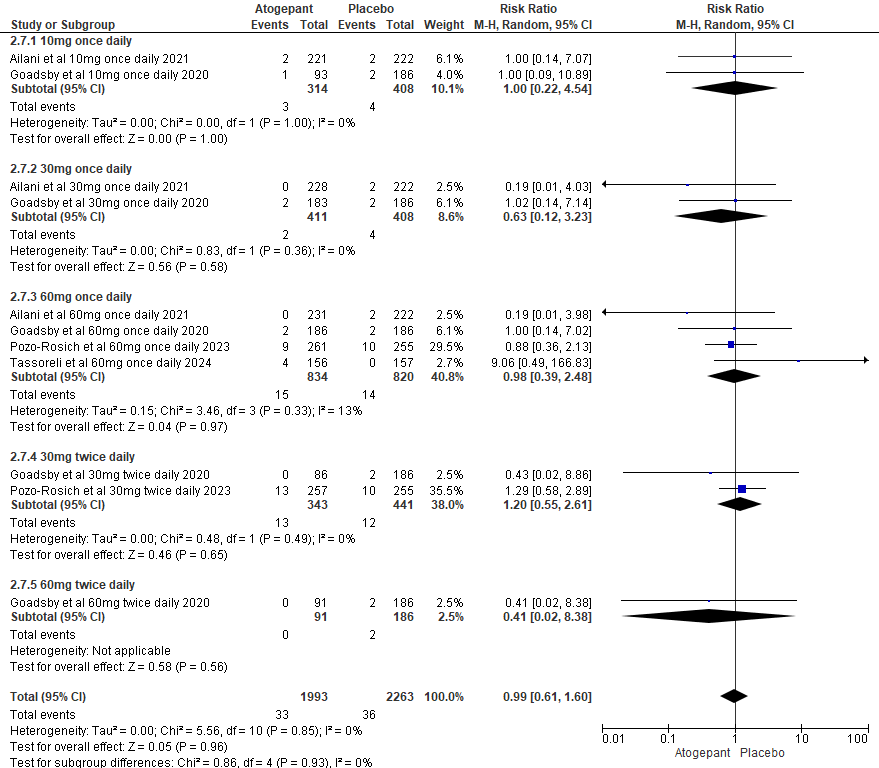
**

**Supplementary Figure 9H:** Score on Role Function–Restrictive domain of MSQ at 12 weeks

**
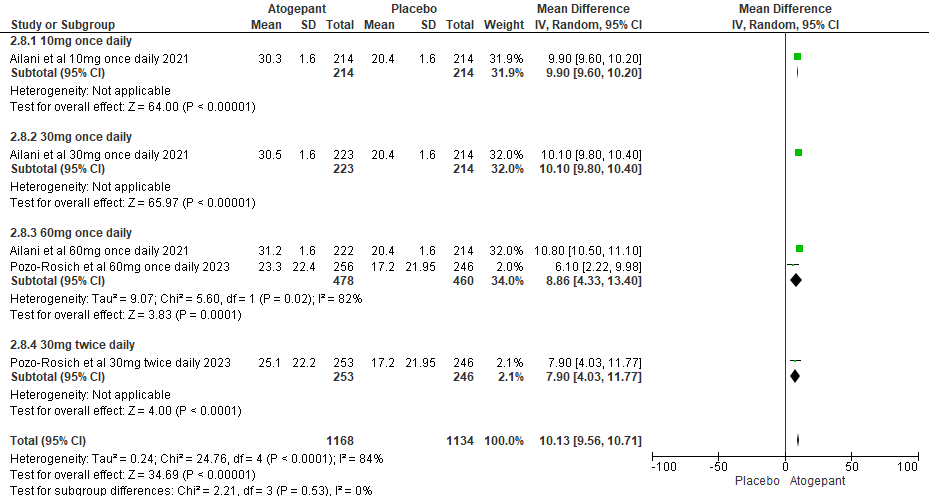
**

**Supplementary Figure 9I:** Score on Performance of Daily Activities domain of AIM-D at 12 weeks

**
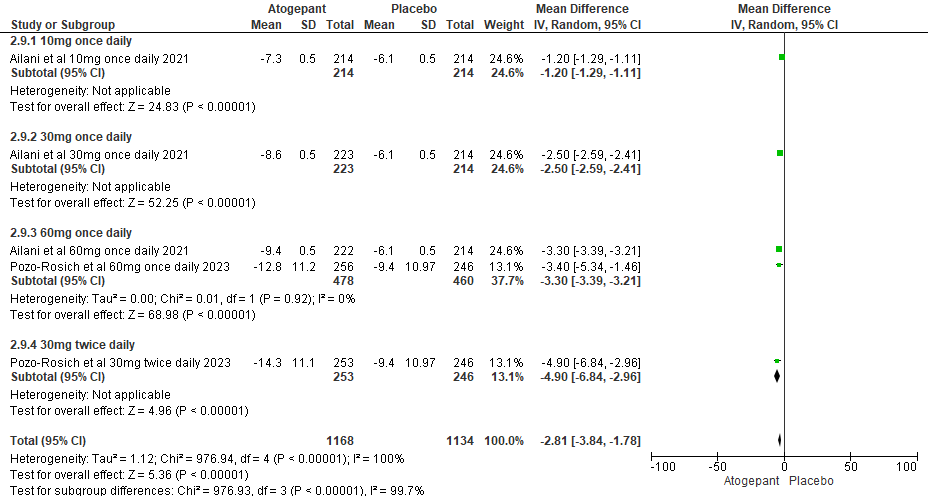
**

**Supplementary Figure 9J:** Score on Physical Impairment domain of AIM-D at 12 weeks

**
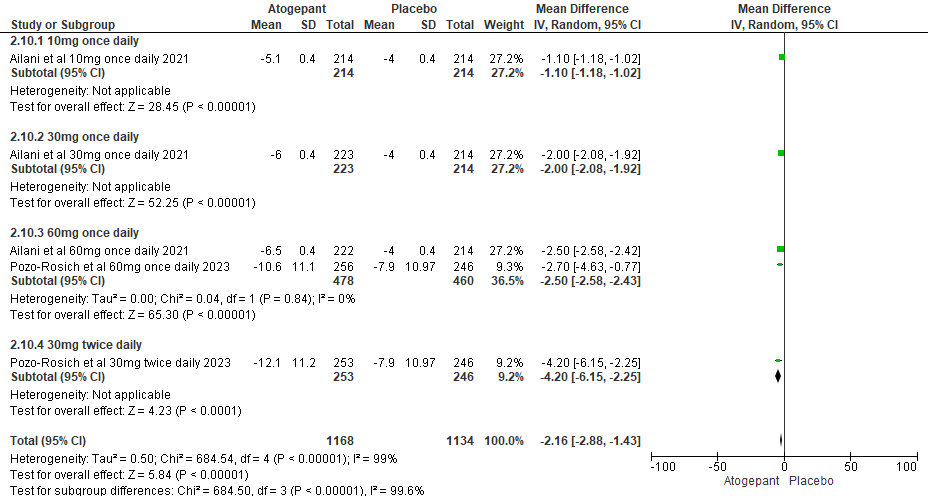
**

**Supplementary Figure 10A:** Mean age, Coeff -0.4448, p value 0.0185

**Supplementary Figure 10B:** Male sex %, Coeff 0.1053, p value 0.1535


**Supplementary Figure 10C:** BMI, Coeff 0.1951, p-value 0.0280

**Supplementary Figure 10D:** Duration of migraine, Coeff -0.0142, p value 0.9838

.
